# Different patterns of antimicrobial non-susceptibility of the nasopharyngeal carriage of *Streptococcus pneumoniae* in areas with high and low levels of PCV13 coverage

**DOI:** 10.1016/j.vaccine.2025.127455

**Published:** 2025-08-30

**Authors:** Jian Wang, Wei Zhao, Shuang Bai, Ao Zhang, Junnan Zhang, Wenwen Lan, Yihan Zhang, Jing Li, Shanshan Zhou, Qun Zheng, Luodan Suo, Min Lv, Jiang Wu

**Affiliations:** a Beijing Center for Disease Prevention and Control, Beijing, China; b Beijing Research Center for Respiratory Infectious Diseases, Beijing, China

**Keywords:** Antimicrobial nonsusceptibility, Streptococcus pneumoniae, Vaccine serotypes, PCV13 coverage

## Abstract

**Introduction:**

The impact of pneumococcal conjugate vaccines (PCVs) on antimicrobial resistance (AMR) in *Streptococcus pneumoniae* (*Spn*) among Chinese children remains inadequately characterized. Evidence is also limited on the interaction between vaccination coverage and regional disparities in AMR patterns. This study aims to assess the impact of the 13-valent pneumococcal conjugate vaccine (PCV13) on nasopharyngeal carriage of *Spn* and antimicrobial nonsusceptibility to commonly used antibiotics among healthy children under five years of age in China.

**Methods:**

In 2022, 737 pneumococci were isolated from 2333 healthy children recruited in 4 areas (Haikou, Wanning, Baisha, and Qiongzhong) in Hainan Province, China. Serotyping and antimicrobial susceptibility tests were performed. Participants were divided into 4 groups based on individual PCV13 vaccination history and the local vaccination coverage rates: vaccinated and unvaccinated groups in the high-coverage area (group VH & NVH), and vaccinated and unvaccinated groups in the low-coverage areas (group VL & NVL). Descriptive statistics and logistic regression analysis were used to assess the vaccine impacts.

**Results:**

PCV13-vaccinated children exhibited significantly lower carriage rates of vaccine-type serotypes (VTs) compared to unvaccinated children: 6B (7.0 % vs. 2.7 %, P < 0.01), 6A (4.2 % vs. 1.2 %, P < 0.05), and 23F (2.2 % vs. 0.3 %, P < 0.05). The non-susceptibility rates to erythromycin, azithromycin, clindamycin, tetracycline, penicillin, and cefuroxime were 92.3 %, 87.5 %, 81.2 %, 91.2 %, 38.9 % and 64.7 %, respectively, resulting in 82.9 % of isolates being multidrug-resistant (MDR), especially to VTs (92.4 %). Isolates from the high PCV13 coverage area were less resistant to penicillin, cefuroxime, erythromycin, azithromycin, clindamycin and SXT, and were less MDR than isolates from the low PCV13 coverage areas.

**Conclusions:**

Nasopharyngeal carriage of *Spn* was highly resistant to common clinical antibiotics. PCV13 vaccination significantly decreased VTs carriage and antimicrobial nonsusceptibility. These results suggest that the widespread use of PCVs is likely to substantially affect NP carriage and antimicrobial nonsusceptibility.

## Highlights

• Children administered PCV13 had a lower carriage rate of vaccine serotypes.
• *Spn* carriage exhibits 82.9% multidrug resistance (MDR), particularly in vaccine serotypes.
• Higher PCV13 coverage linked to lower antibiotic resistance and MDR

## 1 Introduction

Pneumococcal disease is a leading cause of morbidity and mortality among children under 5 years of age. The rising antimicrobial resistance (AMR) of *Streptococcus pneumoniae* (*Spn*) is a serious concern worldwide [1,2]. The disease burden of pneumococcal infections has increased in many countries owing to the emergence of multidrug-resistant (MDR) isolates, particularly in Asia. [3]. *Spn* is a common commensal bacterium found in the upper respiratory tract. Nasopharyngeal (NP) carriage of *Spn* is a predisposing factor for the development of pneumococcal disease. NP carriage of pneumococci is usually asymptomatic. Occasionally, colonizing pneumococci spread from the nasopharynx to the surrounding tissues or invade the bloodstream, leading to pneumococcal mucosal infections and severe invasive pneumococcal disease (IPD) [4]. Therefore, knowledge regarding the AMR of the NP carriage of *Spn* is useful in guiding the clinical use of antimicrobials.

The pneumococcal 13-valent conjugated vaccine (PCV13) was licensed in China in 2016, with a recommended schedule for infants of 3 + 1 doses at 2, 4 and 6 months, followed by a booster at 12–15 months. In China, the PCV13 remains a voluntary immunization option outside the National Immunization Program (NIP), requiring informed consent prior to administration. The complete four-dose vaccination entails out-of-pocket expenses of 1900–2800 Chinese Yuan (CNY) (approximately 260–390 USD). Data retrieved from the provincial Immunization Information System indicated that in 2021, across 9 provinces in China, 35.4 % of infants had received at least one dose of the PCV13 vaccine, while 16.1 % and 12.5 % had completed the full primary series and received a booster dose, respectively [5]. Despite the coverage rate increased annually, the high price contributed to the low affordability in central and western provinces as well as rural areas of China. The implementation of pneumococcal conjugate vaccines (PCVs) has led to a significant reduction in the incidence of IPD and carriage of vaccine-type serotypes (VTs) [6,7]. Some Research conducted in China have demonstrated that administration of the PCV7 or PCV13 vaccines is correlated with a diminished VTs colonization [8,9]. By reducing NP carriage and transmission of antibiotic-resistant VTs pneumococci, PCVs decrease the incidence of pneumococcal infections requiring antibiotic treatment [[10], [11], [12]]. This reduction in antibiotic use, in turn, lowers both demand and misuse, alleviating selective pressure and thereby mitigating the emergence of antimicrobial resistance. For example, following the introduction of PCV7 and PCV13 in the United States and Israel, antibiotic-nonsusceptible IPD incidence and NP carriage were significantly reduced [13,14]. A study offered a comprehensive and quantitative model predicting that introducing PCVs in national immunization program would likely slow the development of AMR in China [15]. However, the benefits of PCVs in relation to the AMR of the NP carriage of pneumococci in China have not been reported.

Previously, we conducted an NP carriage study in Hainan Province. Pneumococcus was isolated from 710 (30.4 %) out of 2333 children under 5 years of age enrolled from four different areas (Haikou, Wanning, Baisha, and Qiongzhong) [9]. We found that Haikou, which had the highest number of children vaccinated with PCV13, had the lowest PCV13 serotype (VTs) carriage rate compared to that of other areas. This observation prompted a post hoc analysis of this study, which examines the impact of PCV13 on NP carriage of *Spn* and antimicrobial nonsusceptibility among children under five years of age in Hainan Province, China, specifically focusing on regional disparities in vaccination coverage.

## 2 Materials and methods

### 2.1 Study design

This study was part of a cross-sectional study of NP carriage of *Spn* in healthy children under 5 years of age, conducted from March to June 2022 in Hainan Province, China. Detailed information on the study design and population has been published previously [9]. Briefly, participants were healthy children aged younger than 59 months who lived in the 4 different areas of Hainan Province: Haikou, Wanning, Baisha, and Qiongzhong. Children were excluded if they had upper or lower respiratory illness or a documented febrile episode within the last 24 h (axillary temperature of ≥ 37.3 °C), if they had congenital malformation or injury of the nasopharynx that would prevent the taking of an NP swab. Children were classified as vaccinated if they had received at least one dose of the PCV13 more than 2 weeks prior to sample collection. Conversely, children were classified as unvaccinated if they had not received any doses of PCV13 prior to sample collection. Among all the healthy children included in the study, 327 children received 1–4 doses of PCV13. Among them, 30.5 % (233/764) and 6.0 % (94/1569) received PCV13 in Haikou and the other three areas (Wanning, Baisha, and Qiongzhong), respectively. A total of 737 *Spn* isolates (2 serotypes isolated from a single specimen in 27 children) from 710 children were included in this analysis. Of the 737 pneumococci, 29 serotypes were identified; 60.9 % (449/737) were PCV13 serotypes (VTs) and 39.1 % (288/737) were non-vaccine serotypes (NVTs) [9]. Subsequently, we investigated the impact of PCV13 on pneumococcal carriage and antimicrobial nonsusceptibility. The study protocol and corresponding informed consent were approved by the Ethics Committee of the Beijing Center for Disease Prevention and Control (No. 202116). Written informed consent was obtained from parents or legal guardian prior to any study procedure.

### 2.2 Study Setting

The study was conducted in 4 areas of Hainan Province: Haikou, Wanning, Baisha, and Qiongzhong. These areas were selected to reflect socioeconomic and healthcare disparities: Haikou (the provincial capital, a high PCV13 coverage area); Wanning, Baisha, and Qiongzhong (low PCV13 coverage areas).

### 2.3 Collection of bacterial isolates and culture conditions

NP sample collection and laboratory tests were performed as described previously [9]. The pneumococcal isolates were stored at −80 °C in skim milk-tryptone-glucose-glycerol(STGG) medium after identification using standard methods [16]. All isolates were cultured on blood agar plates for 18 h at 37 °C in 5 % CO_2_ and the serotypes were confirmed by performing the Quellung reaction with antisera from the Staten Serum Institute (Copenhagen, Denmark).

### 2.4 Antimicrobial susceptibility testing

To prepare the inoculum, all strains were resub-cultured to ensure purity and viability. The micro-dilution method was used to determine the minimum inhibitory concentrations of all *Spn* isolates against 17 antibiotics: penicillin (PEN), amoxicillin (AMX), amoxicillin/clavulanic acid (AMC), cefuroxime (CXM), ceftriaxone (CRO), cefepime (FEP), erythromycin (ERY), azithromycin (AZM), meropenem (MEM), vancomycin (VAN), levofloxacin (LEV), moxifloxacin (MXF), trimethoprim/sulfamethoxazole (SXT), chloramphenicol (CHL), clindamycin (CLI), linezolid (LZD) and tetracycline (TET). Erythromycin-resistant and clindamycin-susceptible or intermediate strains were tested for inducible clindamycin resistance using broth microdilution before reporting clindamycin. The Clinical and Laboratory Standards Institute (CLSI) 2023 clinical breakpoints for *Spn* were applied to classify isolates as susceptible, intermediate, or resistant.

Multi-drug resistance (MDR) was defined as resistance to three or more of the following 11 classes of antibiotics: β-lactams, macrolides (MAC), carbapenems, glycopeptides, fluoroquinolones, folate pathway antagonists, phenicols, lincosamides, oxazolidinones and tetracyclines. Isolates that are resistant to one antibiotic are considered to be resistant to that class of antibiotic. *Spn* ATCC 49619 was used as the quality control strain and was included in each set of tests to ensure accurate results.

### 2.5 Statistical methods

To evaluate both the direct and indirect vaccination effects on antimicrobial nonsusceptibility, participants were stratified into 4 groups based on their vaccination status with regional coverage levels:

- Group VH: Vaccinated children in the high-coverage area.

- Group NVH: Unvaccinated children in the high-coverage area.

- Group VL: Vaccinated Children in the low-coverage areas.

- Group NVL: Unvaccinated children in the low-coverage areas.

ANOVA was used to calculate and compare mean age in different groups. The Chi-square test was used for intergroup comparisons of categorical variables. If the predicted value in any cell was < 5, Fisher's exact test was used. Differences between groups were considered significant at P < 0.05. Odds ratios and 95 % confidence intervals were calculated for the risk of carrying non-susceptible strains in the VH, NVH, VL and NVL groups. Analyses were performed using SPSS version 21.0 (IBM Corp., Armonk, NY, USA) and WPS Office.

## 3 Results

### 3.1 Study participants

The background characteristics of the 710 children are summarized in Table 1. Of these, 41, 17, 172, and 480 children were assigned to the VH, VL, NVH, and NVL groups, respectively. Correspondingly, 43 (VTs = 16, NVTs = 27), 19 (VTs = 10, NVTs = 9), 180 (VTs = 93, NVTs = 87), and 495 (VTs = 330, NVTs = 165) pneumococci were isolated from each group, respectively.

**Table 1:** Background characteristics of healthy children positive for nasopharyngeal carriage in Hainan Province.

Variable, n (%)	Vaccinated (n = 58)		Unvaccinated (n = 652)		Total
	Group VH (n = 41)	Group VL (n = 17)	Group NVH (n = 172)	Group NVL (n = 480)	(n = 710)
**Demographics**					
**Age (months), median (IQR)**	29	36	39	38	38
	(19–43)	(23–45)	(21–51)	(20–51)	(20–50)
**Age group,**					
< 12 months	5 (12.2)	3 (17.6)	21 (12.2)	65 (13.5)	94 (13.2)
12–23 months	10 (24.4)	2 (11.8)	25 (14.5)	80 (16.7)	117 (16.5)
24–35 months	9 (22.0)	3 (17.6)	28 (16.3)	72 (15.0)	112 (15.8)
36–47 months	13 (31.7)	6 (35.3)	46 (26.7)	119 (24.8)	184 (25.9)
≥ 48 months	4 (9.8)	3 (17.6)	52 (30.2)	144 (30.0)	203 (28.6)
**Gender**					
Male	20 (48.8)	8 (47.1)	97 (56.4)	254 (52.9)	379 (53.4)
Female	21 (51.2)	9 (52.9)	75 (43.6)	226 (47.1)	331 (46.4)
**Gestational age**					
Premature	1(2.4)	1 (5.9)	10 (5.8)	21 (4.4)	33 (4.6)
Postmature	40(97.6)	16 (94.1)	162 (94.2)	459 (95.6)	677 (95.4)
**Birth weight, n (%)**					
<2.5 kg	1(2.4)	1 (5.9)	11 (6.4)	37 (7.7)	50 (7.0)
2.5 kg- < 4 kg	39(95.1)	16 (94.1)	160 (93.0)	428 (89.2)	643 (90.6)
≥4 kg	1(2.4)		1 (0.6)	15 (3.1)	17 (2.4)
**Maternal age at enrollment, year**					
<35	33(80.5)	17 (100)	147 (85.5)	421 (87.7)	618 (87.0)
≥35	8(19.5)	0 (0)	25 (14.5)	59 (12.3)	92 (13.0)
**Household registration**					
Local	33(80.5)	16 (94.1)	112 (65.1)	451 (94.0)	612 (86.2)
Stranger	8(19.5)	1 (5.9)	60 (34.9)	29 (6.0)	98 (13.8)
**Breastfeeding (age ≤ 24 months)**					
Never breastfed	2(4.9)	0 (0)	6 (3.5)	24 (5.0)	32 (4.5)
Breastfed before	9(22.0)	3 (17.6)	30 (17.4)	104 (21.7)	146 (20.6)
Still breastfed	5(12.2)	2 (11.8)	14 (8.1)	29 (6.0)	50 (7.0)
**Daycare attendance**					
No	28(68.3)	8 (47.1)	84 (48.8)	225 (46.9)	345 (48.6)
Yes	13(31.7)	9 (52.9)	88 (51.2)	255 (53.1)	365 (51.4)
**Presence of siblings**					
No	8 (19.5)	7 (41.2)	62 (36.0)	125 (26.0)	202 (28.5)
Yes	33(80.5)	10 (58.8)	110 (64.0)	355 (74.0)	508 (71.5)
**Household characteristics**					
**Household tobacco exposure**					
No	22 (53.7)	6 (35.3)	58 (33.7)	122 (25.4)	208 (29.3)
Yse	19 (46.3)	11 (64.7)	114 (66.3)	358 (74.6)	502 (70.7)
**Average living space**					
<10 m^2	2 (4.9)	2 (11.8)	23 (13.4)	14 (2.9)	41 (5.8)
10-20 m^2	17 (41.5)	4 (23.5)	81 (47.1)	200 (41.7)	302 (42.5)
>20 m^2	22 (53.7)	11 (64.7	68 (39.5)	266 (55.4)	367 (51.7)
**Residential area**					
Urban area	29 (70.7)	6 (35.3)	123 (71.5)	44 (9.2)	202 (28.5)
Rural area	12 (29.3)	11 (64.7)	49 (28.5)	436 (90.8)	508 (71.5)
**Socioeconomic status**					
Educational degree of mother					
Elementary / Junior High School	11 (26.8)	6 (35.3)	59 (34.3)	273 (56.9)	349 (49.2)
Senior High / Technical Secondary School	12 (29.3)	2 (11.8)	63 (36.6)	115 (24.0)	192 (27.0)
Junior College / Bachelor / Master	18 (43.9)	9 (52.9)	50 (29.1)	92 (19.1)	169 (23.8)
Educational degree of father					
Elementary / Junior High School	13 (31.7)	3 (17.6)	52 (30.2)	260 (54.2)	328 (46.2)
Senior High / Technical Secondary School	7 (17.1)	2 (11.8)	63 (36.6)	119 (24.8)	191 (26.9)
Junior College / Bachelor / Master	21 (51.2)	12 (70.6)	57 (33.1)	101 (21.0)	191 (26.9)
Per capita monthly disposable income					
<3000 CNY (415 $)	9 (22.0)	7 (41.2)	43 (25.0)	229 (47.7)	288 (40.6)
3000–5999 CNY (415–831 $)	24 (58.5)	7 (41.2)	85 (49.4)	173 (36.0)	289 (40.7)
≥ 6000 CNY (831 $)	8 (19.5)	3 (17.6)	44 (25.6)	78 (16.3)	133 (18.7)
**PCV13 vaccination history**					
0 dose	0 (0)	0 (0)	172 (100)	480 (100)	652 (91.7)
1–2 doses	20 (48.8)	10 (58.8)	0 (0)	0 (0)	30 (4.2)
≥ 3 doses	21 (51.21)	7 (41.2)	0 (0)	0 (0)	28 (3.9)

### 3.2 Serotype distribution in vaccinated and unvaccinated groups

**Fig. 1:**
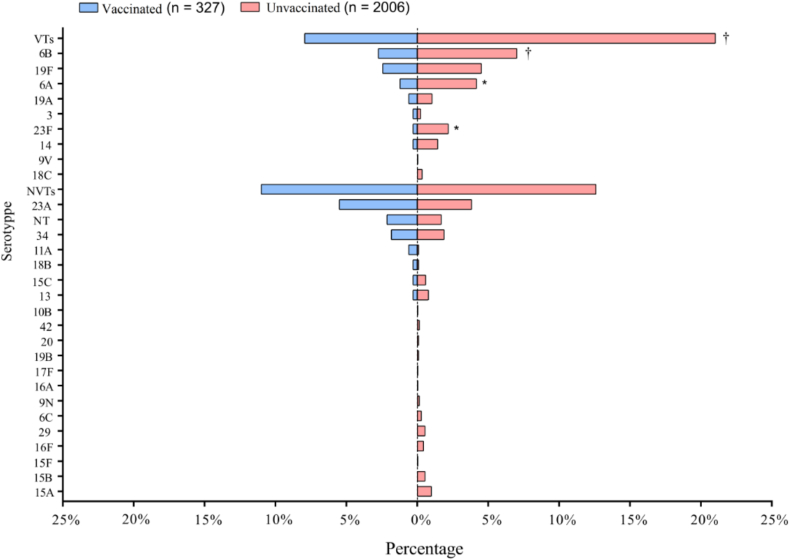
The serotype distribution of *Spn* isolates in vaccinated (blue, children completed 1–4 doses of PCV13) and unvaccinated children (pink, children did not receive PCV13). VTs, vaccine serotypes; NVTs, nonvaccine serotypes; NT, non-typeable (NT) pneumococci. *P < 0.05, †P < 0.01. (For interpretation of the references to colour in this figure legend, the reader is referred to the web version of this article.)

The serotype distribution of pneumococcal isolates was shown in Fig. 1 and supplementary table 1. The serotype distribution was similar between the two groups. Among 2333 isolates, VTs accounted for 19.2 % (449/2333), predominantly 6B (6.4 %, 150/2333), 19F (4.2 %, 99/2333), 6A (3.8 %, 88/2333) and 23F (1.9 %, 45/2333). The most common NVTs (12.3 %, 288/2333) were 23A (4.1 %, 95/2333), 34 (1.9 %, 44/2333) and non-typeable (NT) pneumococci (1.8 %, 41/2333). Compared to those of the unvaccinated group, the vaccinated group had significantly lower carriage rates for VTs (21.0 % vs. 8.0 %, P < 0.001), 6B (7.0 % vs. 2.7 %, P < 0.01), 6 A (4.2 % vs. 1.2 %, P < 0.05), and 23F (2.2 % vs. 0.3 %, P < 0.05).

### 3.3 Antimicrobial non-susceptibility

The antibiotic susceptibilities of the 737 *Spn* isolates to 17 antimicrobials are presented in Table 2. Overall, 38.9 %, 6.8 %, and 6.9 % of the isolates were non-susceptible to penicillin, amoxicillin and amoxicillin-clavulanate, respectively. The non-susceptibility rates for cefuroxime, ceftriaxone, and cefepime were 64.7 %, 10.2 %, and 0.5 %, respectively. The rate of non-susceptibility to macrolides was high, at 92.3 % and 87.5 % for erythromycin and azithromycin, respectively. The rates of non-susceptibility to clindamycin and tetracycline were 81.1 % and 91.2 %, respectively. The non-susceptibility rates to levofloxacin and chloramphenicol were 0.1 % and 5.8 %, respectively. All the isolates were susceptible to vancomycin, levofloxacin, moxifloxacin, and linezolid. No statistically significant differences in non-susceptibility rate were observed across different age groups. The proportion of isolates with intermediate resistance to penicillin (24.3 %), meropenem (22.3 %), and SXT (30.4 %) was high (Supplementary table 2).

**Table 2:** Antimicrobial susceptibility of the 737 *Spn* isolates from children under 5 years of age in Hainan Province.

Antimicrobial Agent		Non-susceptible, n (%)				P value⁎
		0–6 months (n = 31)	7–23 months (n = 190)	≥24 months (n = 516)	Total (n = 737)	
β-lactams	Penicillin (non-meningitis)	15 (48.4)	69 (36.3)	203 (39.3)	287 (38.9)	0.414
	Amoxicillin (non-meningitis)	3 (9.7)	10 (5.3)	37 (7.2)	50 (6.8)	0.489
	Amoxicillin-clavulanate(non-meningitis)	3 (9.7)	10 (5.3)	38 (7.4)	51 (6.9)	0.475
	Cefuroxime	20 (64.5)	120 (63.2)	337 (65.3)	477 (64.7)	0.851
	Ceftriaxone (non-meningitis)	2 (6.5)	17 (8.9)	56 (10.9)	75 (10.2)	0.703
	Cefepime (non-meningitis)	0	0	4 (0.78)	4 (0.5)	0.645
Macrolides	Erythromycin	27 (87.1)	174 (91.6)	479 (92.8)	680 (92.3)	0.387
	Azithromycin	26 (83.9)	167 (87.9)	452 (87.6)	645 (87.5)	0.780
Carbapenems	Meropenem	5 (16.1)	38 (20.0)	123 (23.8)	166 (22.5)	0.423
Glycopeptides	Vancomycin	0	0	0	0	1.000
Fluoroquinolones	Levofloxacin	0	1 (0.5)	0	1 (0.1)	0.300
	Moxifloxacin	0	0	0	0	1.000
Folate pathway antagonists	Trimethoprim-sulfamethoxazole	19 (61.3)	121 (63.7)	360 (69.8)	500 (67.8)	0.209
Phenicols	Chloramphenicol	2 (6.5)	11 (5.8)	30 (5.8)	43 (5.8)	0.957
Lincosamides	Clindamycin	22 (71.0)	154 (81.1)	422 (81.8)	598 (81.1)	0.317
Oxazolidinones	Linezolid	0	0	0	0	1.000
Tetracyclines	Tetracycline	29 (93.6)	171 (90.0)	472 (91.5)	672 (91.2)	0.780

⁎ P-values indicate statistical differences between the 3 age groups.

Of the 737 isolates, 11 (1.5 %) were susceptible to all the antimicrobials, 75 (10.2 %) were resistant to only one class, 40 (5.4 %) were resistant to two classes, and 611 (82.9 %) were resistant to three or more classes. The most common MDR patterns were β-lactams+ MAC + CLI + TET (166, 22.5 %), MAR + CLI + TET (142, 19.3 %) and β-lactams + MAC + CLI+ TET (138, 18.7 %) (Supplementary table 3).

### 3.4 VTs and NVTs related to antibiotic non-susceptibility

**Fig. 2:**
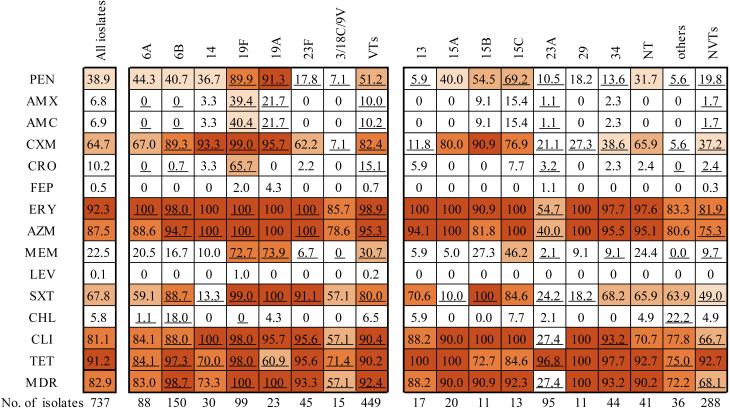
Antimicrobial non-susceptibility and MDR rates of *Spn* isolates in this study. Numbers shown are the antimicrobial non-susceptibility rates or MDR rates of each serotype, or pooled PCV13, or non-PCV13 serotypes. Colored fields indicate a high non-susceptibility rate for the corresponding serotype. Darker colors indicate a higher non-susceptibility rate. Underscored numbers indicate a statistically significant difference in the non-susceptibility rate or MDR of a serotype compared to that of all other serotypes. PEN, penicillin; AMX, amoxicillin; AMC, amoxicillin/clavulanic acid; CXM, cefuroxime; CRO, ceftriaxone; FEP, cefepime; ERY, erythromycin; AZM, azithromycin; LEV, levofloxacin; SXT, trimethoprim/sulfamethoxazole; CHL, chloramphenicol; CLI, clindamycin; TET, tetracycline; MDR, multi-drug resistance. VTs, PCV13 serotypes; NVTs, non-vaccine serotypes.

Among the 737 isolates, 449 (449/737, 60.9 %) belonged to the PCV13 serotype. Fig. 2 shows the rates of antimicrobial non-susceptibility to partial antimicrobials. Non-susceptibility rates varied between serotypes and were higher among the VTs and NVTs 15B and 15C. Among the VTs, 92.4 % of the isolates were MDR, and the rates of non-susceptibility to penicillin, cefuroxime, erythromycin, azithromycin, meropenem, and SXT were the highest in the serotypes 19 A (91.3 %, 95.7 %, 100 %, 100 %, 72.7 % and 100 %, respectively) and 19F (89.9 %, 99.0 %, 100 %, 100 %, 73.9 % and 99.0 % respectively), and the highest rate of non-susceptibility to ceftriaxone was observed in serotype 19 A (65.7 %). Among the NVTs, high rates of non-susceptibility to erythromycin, azithromycin, clindamycin, and tetracycline were observed, except for serotype 23 A. The non-susceptibility rate to penicillin (69.2 %) was the highest in serotype 15C. High rates of non-susceptibility to cefuroxime were also found in 15B (90.9 %),15C (76.9 %), and NT (65.9 %).

### 3.5 Impact of vaccination on antibiotic non-susceptibility

**Fig. 3:**
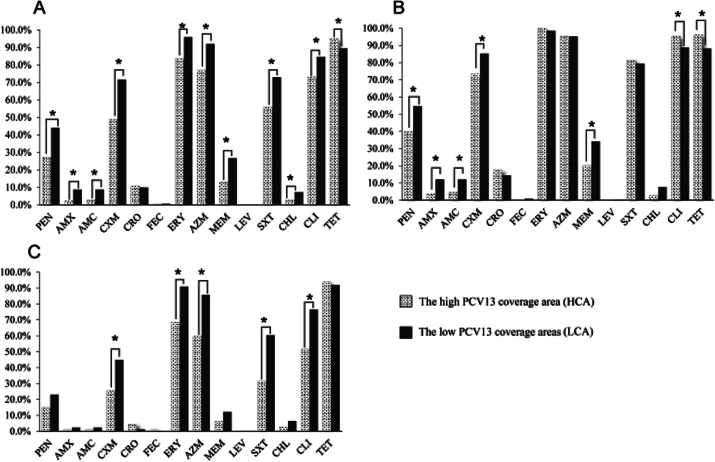
Comparison of antimicrobial non-susceptibility rates of *Spn* isolated from the high PCV13 coverage area (HCA) and the low PCV13 coverage areas (LCA). (A) Antimicrobial non-susceptibility rates of *Spn* from the HCA (n = 223) and LCA (n = 514). (B) Antimicrobial non-susceptibility rates of VTs isolated from the HCA (n = 109) and LCA (n = 340). (C) Antimicrobial non-susceptibility rates of NVTs isolated from the HCA (n = 114) and LCA (n = 174). PEN, penicillin; AMX, amoxicillin; AMC, amoxicillin/clavulanic acid; CXM, cefuroxime; CRO, ceftriaxone; FEP, cefepime; ERY, erythromycin; AZM, azithromycin; LEV, levofloxacin; SXT, trimethoprim/sulfamethoxazole; CHL, chloramphenicol; CLI, clindamycin; TET, tetracycline; VTs, PCV13 serotypes; NVTs, non-vaccine serotypes. *P < 0.05.

Groups VH and NVH were from the high PCV13 coverage area (HCA), and groups VL and NVL were from the low PCV13 coverage areas (LCA). We compared the non-susceptibility patterns of *Spn* isolates from the 2 areas. Fig. 3 shows a comparison of the non-susceptibility of the isolates to partial antimicrobials in the 2 areas. The rates of non-susceptibility to penicillin (27.4 % vs. 44.0 %), amoxicillin (2.2 % vs. 8.8 %), amoxicillin/clavulanic acid (2.7 % vs. 8.8 %), cefuroxime (48.9 % vs. 71.6 %), erythromycin(83.9 % vs. 95.9 %), azithromycin (77.1 % vs. 92.0 %), meropenem (13.0 % vs. 26.7 %), SXT (56.1 % vs. 73.0 %), chloramphenicol (2.7 % vs. 7.2 %), clindamycin (73.1 % vs. 84.6 %), and tetracycline (89.5 % vs. 95.1 %) were significantly lower (P < 0.05) in the HCA, compared with those in the LCA. Additionally, we compared the non-susceptibility patterns of VTs and NVTs isolated from the two areas. Among the VTs, the non-susceptibility rates to penicillin (40.4 % vs. 54.7 %), amoxicillin (3.7 % vs. 12.1 %), amoxicillin/clavulanic acid (4.6 % vs. 12.1 %), cefuroxime (73.4 % vs. 85.3 %) and meropenem (20.2 % vs. 34.1 %) were significantly lower (P < 0.05) in the HCA compared with those in the LCA, whereas the non-susceptibility rates to clindamycin (95.4 % vs. 88.8 %), and tetracycline (96.3 % vs. 88.2 %) were higher (P < 0.05) in the HCA. The rates of non-susceptibility to ceftriaxone, cefepime, erythromycin, azithromycin, or SXT were not significantly different between the two areas. Among the NVTs, the rates of non-susceptibility to cefuroxime (25.4 % vs. 44.8 %), erythromycin (68.4 % vs. 90.8 %), azithromycin (59.6 % vs. 85.6 %), clindamycin (51.8 % vs. 76.4 %), and SXT (31.6 % vs. 60.3 %) were lower (P < 0.05) in the HCA compared with those in the LCA, whereas no significant difference was observed in the rates of non-susceptibility to the other antimicrobials.

**Fig. 4:**
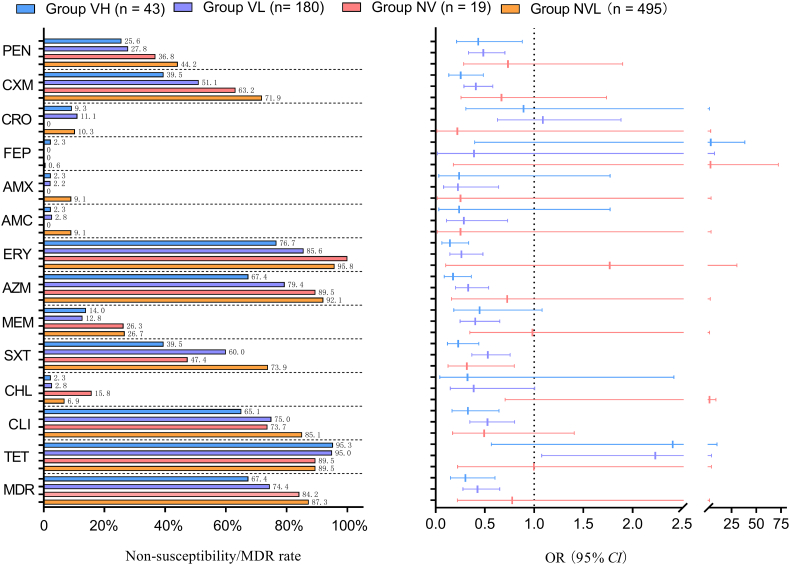
The comparison of the non-susceptibility rates of the isolates to partial antimicrobials and odds ratios (95 % confidence intervals) for the risk of carrying non-susceptible strains in the VH, NVH, VL and NVL groups. PEN, penicillin; AMX, amoxicillin; AMC, amoxicillin/clavulanic acid; CXM, cefuroxime; CRO, ceftriaxone; FEP, cefepime; ERY, erythromycin; AZM, azithromycin; LEV, levofloxacin; SXT, trimethoprim/sulfamethoxazole; CHL, chloramphenicol; CLI, clindamycin; TET, tetracycline; OR, odds ratios; CI, confidence intervals.

Given the impact of PCV13 on antimicrobial nonsusceptibility, we compared the non-susceptibility patterns of *Spn* isolates from the four groups. Fig. 4 shows a comparison of the non-susceptibility rates of the isolates to partial antimicrobials. For most of the antibiotics, there was a significant difference in the rates of non-susceptibility of pneumococcal strains among the four groups, except for ceftriaxone, cefepime, levofloxacin, and tetracycline. Compared with that of NVL group, the VH group had a 57 %–85 % lower risk of carrying strains that were non-susceptible to penicillin (OR [95 %CI] 0.43 [0.21–0.88]), cefuroxime (0.26 [0.13–0.49]), erythromycin (0.15 [0.21–0.88]), azithromycin (0.18 [0.09–0.36]), clindamycin (0.33 [0.17–0.64]), and SXT (0.23 [0.12–0.44]), or were MDR (0.30 [0.15–0.60]). This was also observed for the NVH group for penicillin (OR [95 %CI] 0.49 [0.33–0.70]), amoxicillin (0.23 [0.08–0.64]), amoxicillin/clavulanic acid (0.29 [0.11–0.73]), cefuroxime (0.41 [0.29–0.58]), erythromycin (0.26 [0.14–0.48]), azithromycin (0.33 [0.20–0.54]), clindamycin (0.53 [0.35–0.80]), SXT (0.53 [0.37–0.76]), meropenem (0.40 [0.25–0.65]), chloramphenicol (0.39 [0.15–1.00]), and MDR (0.43 [0.28–0.65]), with a 47 %–77 % reduction in the risk of antimicrobial non-susceptibility (Supplementary table 4).

## 4 Discussion

In the present study, we explored the impact of PCV13 use on NP carriage and antimicrobial non-susceptibility in Hainan Province. We found that children vaccinated with PCV13 had a lower carriage rate of VTs and the *Spn* isolated from the high PCV13 coverage area showed a lower non-susceptibility rate than that from the low PCV13 coverage area.

The implementation of PCVs significantly reduces NP carriage of VTs in individuals [17,18]. Consistent with these findings, our findings revealed that the presence of VTs was lower in children vaccinated with PCV13 than in unvaccinated children. Studies of clinical and NP isolates have revealed a high prevalence of macrolide, clindamycin, and tetracycline resistance in pneumococci in China [[19], [20], [21]]. The data obtained from the current study confirm the high rates of non-susceptibility of pneumococci to these antibiotics. β-lactams, such as penicillin, have traditionally been used to treat infections caused by caused by *Spn*. In this study, the rates of resistance and intermediate resistance to penicillin were 14.5 % and 24.3 %, respectively. The resistance rate of *Spn* to penicillin has increased compared to the 2.2 % resistance rate reported by the Asian Network for Surveillance of Resistant Pathogens in China [21]. Furthermore, a high level of non-susceptibility to cefuroxime (64.7 %) was observed. This finding is similar to those of previous studies conducted in China [22,23].

The increased use of antibiotics has been the primary force driving the emergence and spread of antibiotic-resistant strains in China. β-lactams, macrolides, and clindamycin are commonly used as empirical therapies to treat community-acquired pneumonia and other respiratory tract infections in adults and children [24]. In this study, we found that *Spn* is resistant to common antibiotics, particularly cefuroxime (52.5 %), erythromycin (88.2 %), azithromycin (83.9 %), clindamycin (80.0 %), and tetracycline (88.2 %). Prior research has consistently reported elevated levels of resistance to macrolides, clindamycin, and tetracycline among pneumococci isolates in China and other Asian nations [21,23]. The predominant factors contributing to this heightened prevalence of antimicrobial resistance in Asian regions are attributed to the extensive clinical deployment of these antibiotics and the subsequent clonal dissemination of resistant strains. In contrast, resistance rates to macrolides (39.9 %) and tetracycline (11.0 %) were comparatively lower among children in the United States [25]. A significant proportion of the isolates, 82.9 %, exhibited MDR. This incidence is notably higher than the rates reported in prior studies conducted in Indonesia (18 %), Israel (19.2 %) and Korea (46.2 %) [14,26,27]. The high prevalence of antimicrobial resistance in China is primarily attributed to the widespread use of antibiotics within the primary health care facilities, which has led to a substantial proportion of inappropriate antibiotic prescriptions in these settings. Additionally, the low coverage of PCVs has been insufficient to diminish the prevalence of antibiotic-resistant strains in circulation.

A systematic review of 129 epidemiological studies showed a higher prevalence of non-susceptibility among VTs than among NVTs [28]. Consistent with previous findings, we found that non-susceptibility to some antimicrobials was significantly more common among VT strains, especially the serotypes 19 A and 19F(MDR, 100 %). A previous study suggested that the duration of carrying of a serotype is positively correlated with the prevalence of resistance [29]. Carrying VTs for longer periods and more frequently can cause common and mild mucosal infections in children, such as acute otitis media (AMO). Therefore, VTs are exposed to antibiotics more extensively than NVTs, increasing the opportunity for VT strains to encounter the selective pressure induced by antibiotics. Our recent study showed that the implementation of PCV13 significantly reduced NP carriage of VTs [9], suggesting that the introduction of PCV13 may reduce the proportion of resistant VT strains carried by children. Non-susceptibility to serotypes 15A/B/C and NT in NVTs was also reported in another studies [27,28]. We also identified high non-susceptibility to penicillin, cefuroxime, macrolides, and SXT in 15A/B/C/F and NT. Prior analyses have universally characterized these serotypes as less pathogenic and rare causes of IPD in young children. However, they accounted for a large proportion of the increase in NVTs carriage rates following the implementation of PCVs [30,31]. Thus, the prevalence of non-susceptible NVTs requires monitoring.

In this study, we found that PCV13 vaccination reduced antimicrobial nonsusceptibility, and lower non-susceptibility rates were observed in both vaccinated and unvaccinated groups in areas with high PCV13 coverage rates compared to those in the low coverage areas. The widespread use of PCVs may affect antibiotic-resistant pneumococci through several mechanisms. PCVs can reduce or eliminate the risk of infection of NP carriers caused by antibiotic-resistant VT strains. They also have a secondary effect on AMR by reducing the rates of illness and likelihood of secondary pneumococcal infections [12]. This, in turn, relieves selective pressure induced by antibiotic use, thereby obviating antibiotic misuse. In an observational study conducted in Finland, the percentage of penicillin non-susceptible isolates in children under 5 years of age declined from 25 % to 13 %, and the percentage of multidrug-resistant isolates declined from 22 % to 6 % after PCV10 introduction [32]. Furthermore, a previous study reported that PCV vaccination had significant direct and herd effects reducing VT carriage and antimicrobial non-susceptibility [10]. However, in areas with low PCV13 coverage rates, we found that the isolates did not show lower non-susceptibility rates in the vaccinated group. This suggests that the effects of PCVs on pneumococcal resistance may require a certain level of vaccination coverage to be effective.

Our study has some limitations. First, due to the limited number of strains available, we were unable to stratify our results by the dosage of vaccine received. Consequently, this constraint precluded a more nuanced analysis of the antimicrobial resistance patterns observed. Second, various factors also contribute to antibiotic resistance, such as antibiotic consumption in medical facilities and individual histories of antibiotic exposure. These factors can affect antibiotic resistance patterns, but they were not included in this study. Additionally, there may be differences in the prevalence of antibiotic resistance between pneumococci isolated from patients with invasive diseases and those isolated from the upper respiratory tract.

In summary, the current study provides information on the antimicrobial non-susceptibility patterns of NP carriage of *Spn* among children under 5 years of age in Hainan Province. We observed that pneumococci isolated from areas with higher PCV13 coverage rates exhibited lower drug non-susceptibility even in unvaccinated children, suggesting that increased vaccination rates are likely to indirectly affect NP pneumococcal resistance in children.

## 5 Conclusion

Our findings demonstrate that increased PCV13 coverage reduces VTs pneumococcal carriage and antimicrobial nonsusceptibility. Children, who are particularly vulnerable to pneumococcal diseases, will benefit substantially from the widespread use of PCVs. We advocate for the inclusion of PCV13 in the National Immunization Program, recommending strategies to reduce its cost, expand coverage, address regional disparities, and ensure equitable access to this critical vaccine.

## CRediT authorship contribution statement

**Jian Wang:** Writing – review & editing, Writing – original draft, Formal analysis. **Wei Zhao:** Resources, Investigation. **Shuang Bai:** Resources, Investigation. **Ao Zhang:** Formal analysis. **Junnan Zhang:** Resources, Investigation. **Wenwen Lan:** Resources, Investigation. **Yihan Zhang:** Resources, Conceptualization. **Jing Li:** Formal analysis. **Shanshan Zhou:** Resources, Investigation. **Qun Zheng:** Resources, Investigation. **Luodan Suo:** Project administration. **Min Lv:** Supervision, Funding acquisition, Conceptualization. **Jiang Wu:** Supervision, Funding acquisition, Conceptualization.

## Funding

This work was supported by the Bill & Melinda Gates Foundation (No. INV-034554), Beijing Municipal Natural Science Foundation (No. L202007) and Special funds for the construction of high-level public health technical personnel from Beijing Municipal Health Commission (No. 2022-3-01-021).

## Declaration of competing interest

The authors declare the following financial interests/personal relationships which may be considered as potential competing interests: Jiang Wu reports financial support was provided by Bill & Melinda Gates Foundation. Jian Wang reports financial support was provided by Beijing Municipal Natural Science Foundation. Min Lv reports financial support was provided by Beijing Municipal Health Commission. If there are other authors, they declare that they have no known competing financial interests or personal relationships that could have appeared to influence the work reported in this paper.

## Data Availability

Data will be made available on request.

## Appendix A Supplementary data

Supplementary Tables 1-4 Image 1
