# Supplementary material for: Different patterns of antimicrobial non-susceptibility of the nasopharyngeal carriage of Streptococcus pneumoniae in areas with high and low levels of PCV13 coverage
Source: Vaccine. 2025 Aug 30;62:None. doi: 10.1016/j.vaccine.2025.127455 (PMC12447090; doi:10.1016/j.vaccine.2025.127455)
Supplement: Supplementary file 1 — Supplementary Tables 1-4 [file mmc1.docx]

Supplementary table 1. Serotype distribution in vaccinated and unvaccinated groups

| Serotype | Vaccinated (n=327) | | Unvaccinated (n=2006) | | Total (n=2333) | | *P**-value* |
| --- | --- | --- | --- | --- | --- | --- | --- |
|  | No. | % | No | **%** | No | % |  |
| **VTs** | 26 | 7.95% | 423 | 21.09% | 449 | 19.25% | **0.000** |
| 6B | 9 | 2.75% | 141 | 7.03% | 150 | 6.43% | **0.002** |
| 19F | 8 | 2.45% | 91 | 4.54% | 99 | 4.24% | 0.102 |
| 6A | 4 | 1.22% | 84 | 4.19% | 88 | 3.77% | **0.007** |
| 23F | 1 | 0.31% | 44 | 2.19% | 45 | 1.93% | **0.016** |
| 14 | 1 | 0.31% | 29 | 1.45% | 30 | 1.29% | 0.111 |
| 19A | 2 | 0.61% | 21 | 1.05% | 23 | 0.99% | 0.761 |
| 18C | 0 | 0.00% | 7 | 0.35% | 7 | 0.30% | 0.603 |
| 3 | 1 | 0.31% | 5 | 0.25% | 6 | 0.26% | 0.596 |
| 9V | 0 | 0.00% | 1 | 0.05% | 1 | 0.04% | 1.000 |
| **NVTs** | 36 | 11.01% | 252 | 12.56% | 288 | 12.34% | 0.469 |
| 23A | 18 | 5.50% | 77 | 3.84% | 95 | 4.07% | 0.173 |
| 34 | 6 | 1.83% | 38 | 1.89% | 44 | 1.89% | 1.000 |
| NT | 7 | 2.14% | 34 | 1.69% | 41 | 1.76% | 0.501 |
| 15A | 0 | 0.00% | 20 | 1.00% | 20 | 0.86% | 0.098 |
| 13 | 1 | 0.31% | 16 | 0.80% | 17 | 0.73% | 0.495 |
| 15C | 1 | 0.31% | 12 | 0.60% | 13 | 0.56% | 1.000 |
| 29 | 0 | 0.00% | 11 | 0.55% | 11 | 0.47% | 0.381 |
| 15B | 0 | 0.00% | 11 | 0.55% | 11 | 0.47% | 0.381 |
| 16F | 0 | 0.00% | 9 | 0.45% | 9 | 0.39% | 0.623 |
| 6C | 0 | 0.00% | 6 | 0.30% | 6 | 0.26% | 1.000 |
| 11A | 2 | 0.61% | 2 | 0.10% | 4 | 0.17% | 0.097 |
| 18B | 1 | 0.31% | 2 | 0.10% | 3 | 0.13% | 0.364 |
| 42 | 0 | 0.00% | 3 | 0.15% | 3 | 0.13% | 1.000 |
| 9N | 0 | 0.00% | 3 | 0.15% | 3 | 0.13% | 1.000 |
| 19B | 0 | 0.00% | 2 | 0.10% | 2 | 0.09% | 1.000 |
| 20 | 0 | 0.00% | 2 | 0.10% | 2 | 0.09% | 1.000 |
| 10B | 0 | 0.00% | 1 | 0.05% | 1 | 0.04% | 1.000 |
| 15F | 0 | 0.00% | 1 | 0.05% | 1 | 0.04% | 1.000 |
| 16A | 0 | 0.00% | 1 | 0.05% | 1 | 0.04% | 1.000 |
| 17F | 0 | 0.00% | 1 | 0.05% | 1 | 0.04% | 1.000 |

VTs, PCV13 vaccine serotypes; NVTs, nonvaccine serotypes. Statistically significant differences (*P* ＜ 0.05) are shown in bold.

Supplementary Table 2. Antibiotic profile for 737 *Spn* isolates from children under 5 years of age in Hainan Province

| **Antimicrobial Agent** | | Total （N=737） | | |
| --- | --- | --- | --- | --- |
|  |  | Resistant % | Intermediate % | Susceptible% |
| β-lactams | Penicillin (nonmeningitis) | 14.6% | 24.3% | 61.1% |
|  | Amoxicillin (nonmeningitis) | 0.1% | 6.7% | 93.2% |
|  | Amoxicillin-clavulanat（nonmeningitis) | 0.1% | 6.8% | 93.1% |
|  | Cefuroxime | 52.5% | 12.2% | 35.3% |
|  | Ceftriaxone (nonmeningitis） | 1.8% | 8.4% | 89.8% |
|  | Cefepime (nonmeningitis） | 0.1% | 0.4% | 99.5% |
| Macrolides | Erythromycin | 88.2% | 4.1% | 7.7% |
|  | Azithromycin | 83.9% | 3.6% | 12.5% |
| Carbapenems | Meropenem | 0.3% | 22.2% | 77.5% |
| Glycopeptides | Vancomycin | 0 | 0 | 100.0% |
| Fluoroquinolones | Levofloxacin | 0 | 0.1% | 99.9% |
|  | Moxifloxacin | 0 | 0 | 100.0% |
| Folate pathway antagonists | Trimethoprim-sulfamethoxazole | 37.4% | 30.4% | 32.2% |
| Phenicols | Chloramphenicol | 5.8% | 0.0% | 94.2% |
| Lincosamides | Clindamycin | 80.8% | 0.3% | 18.9% |
| Oxazolidinones | Linezolid | 0 | 0 | 100.0% |
| Tetracyclines | Tetracycline | 88.2% | 3.0% | 8.8% |

Supplementary table 3. Distribution of antibiotic resistance patterns of the Spn isolate

| Antibiotic resistance pattern | No | Percentage |
| --- | --- | --- |
| Susceptible to all the antimicrobials | 11 | 1.5% |
| β-lactams | 1 | 0.1% |
| SXT | 1 | 0.1% |
| MAC | 14 | 1.9% |
| TET | 59 | 8.0% |
| β-lactams/TET | 1 | 0.1% |
| CLI/SXT | 1 | 0.1% |
| MAC/TET | 19 | 2.6% |
| MAC/CLI | 19 | 2.6% |
| MAC/CHL/TET | 1 | 0.1% |
| MAC/CLI/CHL | 1 | 0.1% |
| MAC/SXT/CHL | 1 | 0.1% |
| β-lactams/CHL/TET | 2 | 0.3% |
| SXT/CHL/TET | 2 | 0.3% |
| β-lactams/SXT/TET | 2 | 0.3% |
| β-lactams/MAC/SXT | 2 | 0.3% |
| MAC/SXT/TET | 3 | 0.4% |
| MAC/CLI/SXT | 6 | 0.8% |
| β-lactams/MAC/TET | 13 | 1.8% |
| β-lactams/MAC/CLI | 20 | 2.7% |
| MAC/CLI/TET | 142 | **19.3%** |
| β-lactams/CLI/SXT/TET | 1 | 0.1% |
| β-lactams/MAC/CHL/TET | 1 | 0.1% |
| β-lactams/MAC/SXT/TET | 2 | 0.3% |
| β-lactams/SXT/CHL/TET | 4 | 0.5% |
| MAC/CLI/CHL/TET | 5 | 0.7% |
| β-lactams/MAC/CLI/SXT/ | 10 | 1.4% |
| MAC/CLI/SXT/TET | 61 | 8.3% |
| β-lactams/MAC/CLI/TET | 138 | **18.7%** |
| β-lactams/MAC/SXT/CHL/TET | 2 | 0.3% |
| β-lactams/MAC/CLI/MEM/TET | 2 | 0.3% |
| MAC/CLI/SXT/CHL/TET | 4 | 0.5% |
| β-lactams/MAC/CLI/CHL/TET | 12 | 1.6% |
| β-lactams/MAC/CLI/SXT/TET | 166 | **22.5%** |
| β-lactams/MAC/CLI/SXT/CHL/TET | 8 | 1.1% |
| Total MDR | 611 | **82.9%** |
| Total | 737 | - |

MAC, macrolides; SXT, trimethoprim/sulfamethoxazole; CHL, chloramphenicol; CLI, clindamycin; TET, tetracycline; MDR, multi-drug resistance

Supplementary table 4. The non-susceptibility rates of the isolates to partial antimicrobials and odds ratios (95% confidence intervals) for the risk of carrying non-susceptible strains in the VH, NVH, VL and NVL groups.

| Antimicrobial Agent | Group  VH(n=43) | | |  | | Group NVH  (n=180) | | | |  | | Group VL (n=19) | | | |  | | Group NVL(n=495) **Reference** | |
| --- | --- | --- | --- | --- | --- | --- | --- | --- | --- | --- | --- | --- | --- | --- | --- | --- | --- | --- | --- |
|  | Non-susceptibility | OR (95% CI) | *P* |  | Non-susceptibility | | OR (95% CI) | *P* |  | | Non-susceptibility | | OR (95% CI) | *P* |  | | Non-susceptibility | |  |
| PEN | 25.6% | 0.43(0.21-0.88) | **0.021** |  | 27.8% | | 0.49(0.33-0.70) | **0.000** |  | | 36.8% | | 0.74(0.29-1.9) | 0.525 |  | | 44.2% | |  |
| CXM | 39.5% | 0.26(0.13-0.49) | **0.000** |  | 51.1% | | 0.41(0.29-0.58) | **0.000** |  | | 63.2% | | 0.67(0.26-1.74) | 0.409 |  | | 71.9% | |  |
| CRO | 9.3% | 0.89(0.31-2.60) | 0.835 |  | 11.1% | | 1.09(0.63-1.88) | 0.762 |  | | 0.0% | | 0.22(0.01-3.72) | 0.295 |  | | 10.3% | |  |
| FEP | 2.3% | 3.91(0.4-38.37) | 0.243 |  | 0.0% | | 0.39(0.02-7.58) | 0.543 |  | | 0.0% | | 3.61(0.18-72.29) | 0.401 |  | | 0.6% | |  |
| AMX | 2.3% | 0.24(0.03-1.77) | 0.161 |  | 2.2% | | 0.23(0.08-0.64) | **0.005** |  | | 0.0% | | 0.25(0.02-4.28) | 0.341 |  | | 9.1% | |  |
| AMC | 2.3% | 0.24(0.03-1.77) | 0.161 |  | 2.8% | | 0.29(0.11-0.64) | **0.009** |  | | 0.0% | | 0.25(0.02-4.28) | 0.341 |  | | 9.1% | |  |
| ERY | 76.7% | 0.15(0.06-0.34) | **0.000** |  | 85.6% | | 0.26(0.14-0.73) | **0.000** |  | | 100.0% | | 1.77(0.1-30.25) | 0.694 |  | | 95.8% | |  |
| AZM | 67.4% | 0.18(0.09-0.36) | **0.000** |  | 79.4% | | 0.33(0.2-0.54) | **0.000** |  | | 89.5% | | 0.73(0.16-3.26) | 0.677 |  | | 92.1% | |  |
| MEM | 14.0% | 0.45(0.18-1.08) | 0.074 |  | 12.8% | | 0.4(0.25-0.65) | **0.000** |  | | 26.3% | | 0.98(0.35-2.78) | 0.973 |  | | 26.7% | |  |
| SXT | 39.5% | 0.23(0.12-0.44) | **0.000** |  | 60.0% | | 0.53(0.37-0.76) | **0.001** |  | | 47.4% | | 0.32(0.13-0.8) | **0.015** |  | | 73.9% | |  |
| CHL | 2.3% | 0.32(0.04-2.42) | 0.271 |  | 2.8% | | 0.39(0.15-1.01) | 0.052 |  | | 15.8% | | 2.54(0.71-9.16) | 0.154 |  | | 6.9% | |  |
| CLI | 65.1% | 0.33(0.17-0.64) | **0.001** |  | 75.0% | | 0.53(0.35-0.8) | **0.003** |  | | 73.7% | | 0.49(0.17-1.41) | 0.186 |  | | 85.1% | |  |
| TET | 95.3% | 2.41(0.57-10.24) | 0.235 |  | 95.0% | | 2.23(1.08-4.63) | 0.031 |  | | 89.5% | | 1(0.22-4.44) | 0.998 |  | | 89.5% | |  |
| MDR* | 67.4% | 0.30(0.15-0.60) | **0.001** |  | 74.4% | | 0.43(0.28-0.65) | **0.000** |  | | 84.2% | | 0.78(0.22-2.75) | 0.696 |  | | 87.3% | |  |

*MDR, multidrug-resistant rate

PEN, penicillin; AMX, amoxicillin; AMC, amoxicillin/clavulanic acid; CXM, cefuroxime; CRO, ceftriaxone; FEP, cefepime; ERY, erythromycin; AZM, azithromycin; LEV, levofloxacin; SXT, trimethoprim/sulfamethoxazole; CHL, chloramphenicol; CLI, clindamycin; TET, tetracycline; OR, odds ratios; *CI*, confidence intervals. (*P* ＜ 0.05) are shown in bold.
